# Symptoms, impacts, and suitability of the Pulmonary Arterial Hypertension-Symptoms and Impact (PAH-SYMPACT™) questionnaire in patients with sarcoidosis-associated pulmonary hypertension (SAPH): a qualitative interview study

**DOI:** 10.1186/s12890-021-01694-1

**Published:** 2021-11-12

**Authors:** Brooke M. Currie, Evan W. Davies, Amélie Beaudet, Larissa Stassek, Leah Kleinman, Robert P. Baughman

**Affiliations:** 1 Evidera Inc., Bethesda, MD USA; 2 Actelion Pharmaceuticals Ltd, Allschwil, Basel, Switzerland; 3 Evidera Inc., Seattle, WA USA; 4 University of Cincinnati Medical Center, Cincinnati, OH USA

**Keywords:** Health-related quality of life, PAH-SYMPACT™, Patient-reported outcome, Qualitative interviews, Sarcoidosis, Sarcoidosis-associated pulmonary hypertension

## Abstract

**Background:**

Sarcoidosis-associated pulmonary hypertension (SAPH) is a prevalent and serious complication of sarcoidosis. No SAPH-specific self-report instruments for assessing SAPH symptoms and their impact on patients are available to date. This study sought to determine whether the Pulmonary Arterial Hypertension-Symptoms and Impact (PAH-SYMPACT™) questionnaire is suitable for use in patients with SAPH.

**Methods:**

Patients diagnosed with SAPH participated in qualitative one-on-one telephone interviews to better understand SAPH symptoms and their impacts on patients’ lives and to determine the appropriateness of the PAH-SYMPACT™ for use in patients with SAPH. The interviews comprised concept elicitation, completion of the PAH-SYMPACT™, and cognitive debriefing. Interview transcripts were analyzed by content analysis.

**Results:**

Eleven patients with SAPH were interviewed between August 2019 and June 2020. In the concept elicitation, all 11 participants endorsed shortness of breath and nine participants (82%) rated it as their “most bothersome or severe” symptom. Impacts endorsed by all 11 participants were difficulty walking uphill or up stairs and difficulty in performing daily activities. Cognitive debriefing indicated that the PAH-SYMPACT™ items were relevant and understandable to most participants and reflected their experiences of SAPH. Participants indicated that no key symptoms or impacts of SAPH were missing. They also reported that the PAH-SYMPACT™ instructions and response options were clear, and that it would be feasible to complete the 11 symptom items and one oxygen use item as part of their daily schedule.

**Conclusions:**

This study suggests the PAH-SYMPACT™ is suitable for assessing symptoms and their impact in patients with SAPH. However, larger longitudinal studies are needed to confirm that it is fit for use in this patient population and that it can be used to reliably detect temporal changes in patients’ symptom status.

*Trial registration* Not applicable.

**Supplementary Information:**

The online version contains supplementary material available at 10.1186/s12890-021-01694-1.

## Background

Sarcoidosis is a rare, chronic, multisystemic granulomatous disease of unknown etiology that most commonly affects the lungs and lymphatic system [1–5]. Up to a fifth of patients with pulmonary sarcoidosis develop advanced disease characterized by pulmonary fibrosis [6]. An estimated 6–28% of patients with sarcoidosis [7–9] and up to 74% of patients with advanced pulmonary sarcoidosis suffer from pulmonary hypertension (PH) as a complication [1, 10, 11]. Sarcoidosis-associated PH (SAPH) causes significant morbidity and mortality over and above that caused by sarcoidosis itself [1, 11–14]. In a retrospective cohort study carried out in the United States, nearly three-quarters of patients with SAPH required assistance with daily functional activities [10]. Currently, there is no approved medicinal treatment for SAPH.

Patient-reported outcomes (PROs) can improve understanding of how patients experience sarcoidosis (including SAPH) and its treatment, thus helping to guide disease management [15, 16]. PRO instruments used in clinical trials in sarcoidosis include generic instruments such as the SF-36, non-specific instruments such as the St. George’s Respiratory Questionnaire, and sarcoidosis-specific instruments such as the Sarcoidosis Health Questionnaire, King’s Sarcoidosis Questionnaire (KSQ), and Sarcoidosis Assessment Tool (SAT) [17–20]. To date, no SAPH-specific PROs are available.

The Pulmonary Arterial Hypertension-Symptoms and Impact (PAH-SYMPACT™) [21] questionnaire is a PRO instrument for capturing the symptoms and impacts of pulmonary arterial hypertension (PAH), a subtype of PH [3]. Psychometric validation of the PAH-SYMPACT™ using data from a Phase IIIb trial in patients with PAH indicated that it captured disease symptoms and impacts relevant to patients with PAH, was able to differentiate between patients based on disease severity, and was sensitive to improvements in disease [22].

Patients with SAPH exhibit symptoms similar to those in PAH, including dyspnea and fatigue [22, 23]. This suggested that the PAH-SYMPACT™ might be suitable for assessing symptoms and their impacts in patients with SAPH. The qualitative study described herein was conducted to better understand SAPH symptoms and their impacts on patients’ lives, and to determine the appropriateness of the PAH-SYMPACT™ for use in patients with SAPH.

## Methods

### Study design and participants

Patients diagnosed with SAPH were recruited from two clinical sites in the United States to participate in one-on-one qualitative telephone interviews. During the interviews, which were held between August 2019 and June 2020, participants completed the PAH-SYMPACT™.

The protocol was approved by Ethical and Independent Review Services (approval number 18186). The clinical sites were approved by Ethical and Independent Review Services (approval number 18186–01) and University of Cincinnati institutional review board (approval number 2019–0115). The study was conducted in accordance with the ethical standards of the Declaration of Helsinki (1964) and its subsequent amendments, and with International Council for Harmonisation Good Clinical Practices. All participants gave written informed consent to participate and permitted audio-recording of the interviews.

Eligible participants were English-speaking adults aged 18–85 years, diagnosed with SAPH (confirmed by right heart catheterization or echocardiography), and who were in World Health Organization functional class II or III [24]. Potential participants were excluded if they had severe airway obstruction, PH with an etiology other than sarcoidosis, or pulmonary symptoms primarily attributed to another condition.

### PAH-SYMPACT™

The PAH-SYMPACT™ was developed using patient input to assess symptoms and impacts of PAH [21, 22]. It consists of 23 items: 11 items on symptoms with a 24-h recall period, 11 items on impacts with a 7-day recall period, and one item on oxygen use in the previous 24 h. The symptom items are grouped into cardiopulmonary and cardiovascular domains, and the impact items are grouped into physical and cognitive/emotional domains. To avoid confusion for the SAPH patients who participated in this study and reviewed the questionnaire as part of the interview, references to “PAH” were removed from the title and instructions of the PAH-SYMPACT™.

### Qualitative interviews

The telephone interviews, which lasted approximately 90 min, were conducted by two qualified researchers using a semi-structured qualitative interview guide. The interviews consisted of two parts: concept elicitation and cognitive debriefing. Concept elicitation included open-ended questions to identify SAPH symptoms that were important to participants, as well as the key impacts of symptoms on participants’ lives. Participants were asked to indicate which symptoms were “most bothersome or severe” and which impacts were “most difficult to cope with.” After concept elicitation, participants completed the PAH-SYMPACT™ and were debriefed on the instrument. Participants were asked about the comprehensibility and relevance of the PAH-SYMPACT™ items, and the clarity of the instructions and response options. For a select subset of items, participants were also asked to indicate the level of improvement they would consider to be meaningful. Additional details of the qualitative interview process are provided in Additional file 1: Table S1.

### Analysis

Audio files were transcribed verbatim. Transcripts were reviewed to remove all identifiable information about participants and to correct any obvious transcription errors.

Transcripts were analyzed by content analysis [25, 26] using ATLAS.ti version 8.3 (ATLAS.ti Scientific Software Development GmbH, Berlin) [27]. Qualitative findings were summarized by calculating frequencies and percentages. Descriptive statistics for quantitative data were calculated using SAS 9.4 (SAS Institute Inc., Cary, NC). Additional details of the analysis are provided in Additional file 1: Table S1.

Themes that emerged during concept elicitation were recorded in a saturation grid, which was organized by dividing the transcripts chronologically into four sets of two to three transcripts each. Concepts were considered to have been saturated when the inclusion of additional study participants did not yield any new concepts [28].

## Results

### Participant characteristics

Eleven participants were interviewed (Table 1). Mean participant age was 66.6 years. All participants were female and most were Black or African American (73%). Two-thirds of the participants (64%) had at least some college education. The mean time since diagnosis of sarcoidosis was 23.7 years, and the mean time since confirmation of PH was 5.5 years (Table 2). Eight participants (73%) reported that they were currently using or had previously used oxygen.

**Table 1:** Sociodemographic characteristics

Characteristic	N = 11
Age (years), mean (SD)	66.6 (7.3)
*Gender, n (%)*	
Female	11 (100)
*Ethnicity, n (%)*	
Not Hispanic or Latino	9 (82)
Missing	2 (18)
*Racial background*^*a*^*, n (%)*	
Black or African American	8 (73)
White	3 (27)
*Marital status, n (%)*	
Single	3 (27)
Married	4 (36)
Divorced	4 (36)
*Employment status, n (%)*	
Employed, full-time	1 (9)
Retired	8 (73)
Disabled	2 (18)
*Highest level of education, n (%)*	
Secondary/high school	4 (36)
Some college	2 (18)
College degree	3 (27)
Postgraduate degree	2 (18)

^a^Responses were not mutually exclusive

**Table 2:** Clinical characteristics

Characteristic	N = 11
*Time since diagnosis of sarcoidosis (years), mean (SD)*	23.7 (12.1)
*Time since confirmation of PH (years), mean (SD)*	5.5 (3.8)
*Method used to confirm PH, n (%)*	
Right heart catheterization	9 (82)
Echocardiogram only	2 (18)
*Current treatment for SAPH*^*a*^*, n (%)*	
Anticoagulants	2 (18)
Calcium channel blockers	1 (9)
Diuretics	4 (36)
Endothelin receptor antagonists	6 (55)
Phosphodiesterase type 5 inhibitors	5 (45)
Oxygen	4 (36)
*Current treatment for sarcoidosis*^*a*^*, n (%)*	
Adrenocorticotropin hormone analogue	1 (9)
Bronchodilators	2 (18)
Corticosteroids	8 (73)
Hydroxychloroquine	0
Immunosuppressants (azathioprine)	1 (9)
Monoclonal antibodies (rituximab)	1 (9)
*History of oxygen use*^*b*^	8 (73)
*WHO functional class, n (%)*	
II	6 (55)
III	5 (45)
*FVC (%), mean (SD)*	58.1 (15.7)
*FEV1 (L), mean (SD)*	1.0 (0.4)
*Pulmonary arterial pressure (mmHg), mean (SD)*	35.6 (5.4)
*Comorbid conditions*^*a*^*, n (%)*	
Anemia	2 (18)
Anxiety	4 (36)
Asthma	4 (36)
Atrial fibrillation	4 (36)
Congestive heart failure	2 (18)
Degenerative disk disease	2 (18)
Depression	3 (27)
Diabetes	3 (27)
With chronic complications	1 (9)
Without chronic complications	2 (18)
Gastroesophageal reflux disease	1 (9)
Hypertension	6 (55)
Hypersensitivity lung disease	2 (18)
Obstructive sleep apnea	2 (18)
Osteoporosis	2 (18)
Other health condition(s)^c^	10 (91)
None	0

Authorized personnel at the recruiting sites entered clinical information on the study participants into a case report form

FEV1, forced expiratory volume in the first second; FVC, forced vital capacity; PH, pulmonary hypertension; SAPH, sarcoidosis-associated pulmonary hypertension; WHO, World Health Organization

^a^Responses were not mutually exclusive

^b^Self-reported (other data were captured by personnel at the recruiting sites using a case report form)

^c^Other health conditions, each reported by one participant, were acquired immunoglobulin deficiency, ampullary carcinoma, anticoagulant positive, antiphospholipid syndrome, aortic stenosis, aspergilloma, benign neoplasm of colon, cardiac sarcoidosis, chronic kidney disease, chronic pulmonary heart disease, chronic obstructive pulmonary disease, complete heart block, coronary artery disease, duodenal adenoma, dyslipidemia, esophageal dysphagia, esophageal stricture, fibromyalgia, hypercalcemia, hyperlipidemia, hypothyroid, lupus, Melkersson-Rosenthal syndrome, paroxysmal supraventricular tachycardia, pulmonary embolism, subdural bleeding, supraventricular tachycardia, thrombocytopenia, transient ischemic attack, and uterine cancer

### Concept elicitation

Analysis of the four sets of transcripts indicated that 20 symptom concepts and 25 impact concepts were captured in transcripts 1 and 2 (Additional file 1: Table S2 and Table S3). Three new symptoms and one new impact were captured in transcripts 3 to 5, and one new symptom and five new impacts were captured in transcripts 6 to 8. Aside from three symptom concepts that were judged to overlap with previously reported concepts or to be related to other health conditions, no new symptom or impact concepts were captured in transcripts 9 to 11. This indicated that saturation of concepts had been achieved within the first eight interviews and that no additional interviews were required beyond those that had been conducted.

Symptoms endorsed by participants are shown in Table 3 and representative quotations are provided in Table 4. All participants reported shortness of breath, which worsened with activity and for two participants was worse during the summer. All participants also mentioned swelling in the ankles or legs. Ten participants (91%) reported experiencing fatigue. Lack of energy, wheezing, cough, skin issues, and rapid heartbeat were each reported by nine participants (82%). One participant reported experiencing wheezing almost every day, regardless of activity. Four of the nine participants who reported cough indicated that it could be either dry or wet. In two participants, cough was worse in extreme temperatures or showed seasonal variation. Nine participants (82%) identified shortness of breath as their “most bothersome or severe” symptom, and two participants (18%) selected cough as their “most bothersome or severe” symptom.

**Table 3:** Summary of endorsed symptom concepts

Concept	Endorsement, n (%)	“Most bothersome or severe” symptom, n^a^
Shortness of breath^b^	11 (100)	9
Swelling in ankles or legs^b^	11 (100)	0
Fatigue^b^	10 (91)	0
Lack of energy^b^	9 (82)	0
Wheezing	9 (82)	0
Cough^b^	9 (82)	2
Skin issues	9 (82)	1
Rapid heartbeat^b^	9 (82)	1
Chest pain^b^	8 (73)	0
Eye issues	8 (73)	2
Lightheadedness^b^	8 (73)	0
Non-chest pain	7 (64)	1
Heart palpitations^b^	7 (64)	0
Chest tightness^b^	6 (55)	0
Headache	3 (27)	1
Swelling in stomach area^b^	3 (27)	0
Other symptoms^c^	6 (55)	0

PAH-SYMPACT™: Pulmonary Arterial Hypertension-Symptoms and Impact

^a^Participants could select more than one symptom

^b^Symptom concepts included as items in the PAH-SYMPACT™

^c^Other symptoms, each reported by one participant, were feeling of a lump in the throat/needing to clear the throat (congestion), legs feeling like they will “give out,” numbness in fingers, swelling in hands, problems urinating (due to sarcoidosis in kidneys), balance issues (due to sarcoidosis and pulmonary hypertension), seizures and Bell’s palsy symptoms (due to sarcoidosis in the brain and stress), heavy feeling in the chest/pressure on the chest (different than tightness or pain), weakness (general), weakness and pain in hands, and left side of body feels different (feels “not good”)

**Table 4:** Representative quotations for the most frequently endorsed symptom concepts

Symptom concept	Quotation [participant ID]
Shortness of breath	“Shortness of breath? Out of breath…Sucking for air. You know, I don’t know. I’m just out of breath. I’m not getting enough breath, enough oxygen in or something.” [1–7]
	“Really if I’m walking, like even if I’m in the living room and I have to walk to the bathroom or go to the bathroom, it’s like I’m like [panting], like it’s cutting off down my windpipe by my lungs.” [2–4]
	“…even if I just wash the dishes, it feels like I worked an 8-hours job, and you’ll be so tired. And if you’ve got an appointment, getting dressed, 4 hours. That’s with the clothes out, because it’s going to take that long. Because you’re going to have to stop and rest. And then if you don't stop and rest, your oxygen will go so low you’re going to have to rest until you get at least 97%. I had it go all the way down to 74 one time when I was getting dressed.” [1–3]
Fatigue	“Hmm, just feel kind of wore out [fatigue]. Just feel like you just tired.” [1–11]
	“It’s according to all the activity or what I have to do that determines how fatigued I’m going to be. Like I say, I can go bowl and we’ll bowl from 6:00 in the evening and we’ll get finished about 9:30 or 10:00. […]. I’m okay. But if it’s something that say I have to walk to go somewhere, then walk back and then walk, that tires me out.” [1–7]
Lack of energy	“…Just tired, lack of energy, no motivation.” [1–7]
	“Lack of energy is like you feel like you got the blahs, for me. For me, I feel like I got the blahs, and I’m just soaked out. But fatigue is like you’re just tired. You’ve exerted yourself. You did this walking at work, and you’ve stopped by the store and you darted in here. And you’re just tired. But the lack of energy is like oh, I feel like I can’t – I got to go and get me a cup of coffee. So, that’s what the lack of energy is for me.” [1–11]
Swelling in ankles or legs	“Oh, sometimes it looks like monster’s feet, you know? They’re so swollen that they look like – they don’t look like my feet. And it’s difficult to walk when they’re that swollen.” [1–8]
	“Just holding water [swelling in their ankles]. They say my kidneys ain’t working or something wasn’t working right or something, but my kidneys are good. So, I think it was just the medicine, the prednisone, and the sarcoidosis together.” [1–7]
Wheezing	“[The wheezing is] not always there…But say I’m rushing to do something or I’m going somewhere and I’m getting ready, trying to get stuff together, carrying stuff. […].” [1–7]
	“I can’t describe it. It’s just like a wheezing, like a hay fever or something like that, but I’m congested. You know, it’s like a lot of mucus, cold, and it’ll be down in my chest, and it causes me to wheeze.” [1–11]
Cough	“Oh, god, my cough can be downright terrible, because I’ve coughed so hard sometimes…my whole chest burns…[…]. I would just shake like, […], and I would have a bad coughing spell.” [1–1]
	“It’s typically a dry cough, and it’s just a little nagging thing in the throat that makes it happen. […]. Because, otherwise, it’s kind of a dry cough, but over the years I’ve noticed it’s a deeper, harder cough, yeah. […].” [1–2]
Skin issues	“I started developing like these they looked like blisters on my thighs on both sides…So, I went to a dermatologist, and the dermatologist looked at it, at both of my legs. And I told her they don’t get well…They get a scab, but they come right back.” [1–11]
	“I have some on my legs. They were raised and purplish. I have some on my arm. I have one, it was like a little row of them, and they were raised little bumps. […]. And they just come. I don’t know why, but they just come.” [1–9]

Impacts endorsed by participants are shown in Table 5 and representative quotations are provided in Table 6. All 11 participants reported difficulty walking uphill and/or up stairs, which they primarily attributed to shortness of breath. Ten participants (91%) reported that their general ability to walk was also impaired, again mainly because of shortness of breath. Sixty percent of the participants whose general ability to walk was affected had difficulty walking longer distances (e.g., going out shopping, taking a walk down the block; n = 6), and half (n = 5) had trouble walking even short distances (eg, walking inside a store, walking around the house, going to the bathroom). All 11 participants reported difficulty in performing daily activities, with 10 participants (91%) indicating that their ability to do housework was affected. Six participants (55%) had difficulty with sweeping or vacuuming, four (36%) had difficulty cleaning their house, three each (27%) had problems washing the dishes, doing laundry, dusting, and cooking, and two (18%) had difficulty running errands. Ten participants (91%) reported that their disease impacted their ability to carry things, once again mainly because of shortness of breath. Nine participants (82%) reported difficulties with participating in hobbies or social activities because of their sarcoidosis or SAPH. Three participants (27%) reported that SAPH had affected their ability to drive. The impacts that were most frequently identified as the “most difficult to cope with” were dependence on others (n = 3; 27%), impacts on relationships (n = 2; 18%), parenting or family impacts (n = 2; 18%), and impact on the ability to walk uphill or up stairs (n = 2; 18%) (Table 5).

**Table 5:** Summary of endorsed impact concepts

Concept	Endorsement, n (%)	Impact “most difficult to cope with,” n^a^
*Difficulty walking uphill/up stairs*	11 (100)	2
Stairs	11 (100)	2
Hills^b^	9 (82)	0
*Daily activities overall*	11 (100)	1
Housework^b^	10 (91)	0
Running errands/shopping	2 (18)	0
Driving	3 (27)	0
Other daily activities	7 (64)	0
*Ability to walk (general)*	10 (91)	0
Walking quickly^b^	5 (45)	0
Walking slowly^b^	5 (45)	0
Walking on flat surface^b^	7 (64)	0
*Carrying things*^*b*^	10 (91)	1
*Hobbies or social activities*	9 (82)	1
Hobbies	9 (82)	0
Social activities	1 (9)	0
*Work and volunteering*	8 (73)	0
*Dependence on others*^*b*^	7 (64)	3
*Relationships*	7 (64)	2
*Mental functioning*^*b*^	7 (64)	1
*Self-care activities*^*b*^	7 (64)	0
*Sleep impacted*	7 (64)	0
*Running or exercise*	6 (55)	0
*Feeling frustrated or angry*^*b*^	5 (45)	0
*Feeling worried or anxiety*^*b*^	4 (36)	1
*Feeling sad or depression*^*b*^	4 (36)	1
*Parenting or family impact*	3 (27)	2
*Feeling embarrassed*	3 (27)	0
*Other impacts*^*c*^	8 (73)	1^d^

PAH-SYMPACT™: Pulmonary Arterial Hypertension – Symptoms and Impact; PH: pulmonary hypertension

^a^Participants could select more than one impact

^b^Impact concepts included as items in the PAH-SYMPACT™

^c^Other impacts, each reported by one participant unless otherwise specified, were talking or singing impacted (due to changed voice, difficulty talking with exertion) (n = 2), bone issues (fragile, “dead bone”) (n = 2), clothing or shoe fit from swelling (n = 2), knowing you will never be healthy, weight loss, and incontinence

^d^Knowing you will never be healthy

**Table 6:** Representative quotations for the most frequently endorsed impact concepts

Impact concept	Quotation [participant ID]
Difficulty walking uphill/up stairs	“Walking up and down the steps – I can’t make it up a flight of steps without having to stop to breathe.” [1–4]
	“I can climb up steps, but what I do – because like I’ll go one step at a time, and then I might have to sit down halfway there. […]. And even with my groceries, you know, I’ll go bag by bag…It might take me about half an hour to get up my steps, but I’ll get up my steps, I go step by step…” [1–1]
Daily activities	“Well, anything that’s considered exertion. Like if I’m going to do some vacuuming or mopping, and I don’t do these things super, super often, but if I’m going to do those things, […]. Because the reality is I do notice that if I’m vacuuming the floor, just that continuous movement and force that I’m having to exert, does make me breathe harder.” [1–2]
	“I can’t run to the store…I can’t walk too far and when I’m walking, I get to my destination out of breath. So, if I go to the store, I have to get a cart and lean on the cart until I can compose myself. […]” [1–10]
Ability to walk (general)	“Oh, we can walk slowly with oxygen…And not very long. We have figured out if we go shopping, we go to one store and look for one thing and then leave. [laughs] It’s not like when we used to be able to go shopping all day and have a good time.” [1–4]
	“Well, if I walk less than a block, that’s fatigue for me. Lack of energy. It’s difficult for me to get around. […].” [1–9]
	“Really if I’m walking, like even if I’m in the living room and I have to walk to the bathroom or go to the bathroom, […], like it’s cutting off down my windpipe by my lungs.” [2–4]
Carrying things	“Oh yes. Yes, I do. I have to either leave [grocery bags] in the car or pray someone is at home or call to make sure, especially if it’s something that needs to be refrigerated right away.” [1–9]
Hobbies or social activities	“[…]. I don’t play any sports anymore, and I was a big sports player. I watch it now. No sports at all.” [1–9]
	“…I like to camp and although we can still – I still go camping at times. I’m restricted and to the hiking part of it or you know, all of the things that I want to see. I have to send the kids down with cameras [laughter]. Take pictures of that for me.” [1–8]

### Cognitive debriefing

Following concept elicitation, participants completed the PAH-SYMPACT™ and were then debriefed on the instrument to explore whether it could be used to assess symptoms and impacts in SAPH. Symptoms reported by participants during this part of the interview were based on experiences in the previous 24 h, while reported impacts were based on the previous 7 days.

Based on responses to the PAH-SYMPACT™, seven participants (64%) had used oxygen in the previous 24 h (Table 7). All 11 symptom items had each been experienced by at least four participants (36%) in the previous 24 h. All 11 participants reported shortness of breath, 10 (91%) reported fatigue, and 9 (82%) reported a lack of energy.

**Table 7:** PAH-SYMPACT™ responses

Item	Response, n (%)	
*Oxygen use*	Yes	No
In the past 24 h…		
“Did you use oxygen?”	7 (64)	4 (36)

Item	Experienced	Not experienced
*Symptoms*		
In the past 24 h…		
“How would you rate your shortness of breath?”	11 (100)	0
“How would you rate your fatigue?”	10 (91)	1 (9)
“How would you rate your lack of energy?”	9 (82)	2 (18)
“How would you rate the swelling in your ankles or legs?”	8 (73)	3 (27)
“How would you rate the swelling in your stomach area?”	4 (36)	7 (64)
“How would you rate your cough?”	7 (64)	4 (36)
“How would you rate your heart palpitations (heart fluttering)?”	5 (45)	6 (55)
“How would you rate your rapid heartbeat?”	6 (55)	5 (45)
“How would you rate your chest pain?”	6 (55)	5 (45)
“How would you rate your chest tightness?”	7 (64)	4 (36)
“How would you rate your lightheadedness?”	6 (55)	5 (45)
*Impacts*		
In the past 7 days…		
“Were you able to walk slowly on a flat surface?”	7 (64)	4 (36)
“Were you able to walk quickly on a flat surface?”	10 (91)	1 (9)
“Were you able to walk uphill?”	11 (100)	0
“Were you able to carry things?”	10 (91)	1 (9)
“Were you able to do light indoor household chores?”	8 (73)	3 (27)
“Were you able to wash or dress yourself?”	7 (64)	4 (36)
“How much did you need help from others?”	7 (64)	4 (36)
“Were you able to think clearly?”	7 (64)	4 (36)
“How sad did you feel?”	10 (91)	1 (9)
“How worried did you feel?”	9 (82)	2 (18)
“How frustrated did you feel?”	10 (91)	1 (9)

PAH-SYMPACT™, Pulmonary Arterial Hypertension-Symptoms and Impact

The 11 impact items had each been experienced by at least seven participants (64%) in the previous 7 days (Table 7). All participants had experienced difficulty walking uphill.

Most participants (n = 9; 82%) indicated that all symptom items were relevant to their experiences of sarcoidosis or SAPH, even if they had not experienced a given symptom in the previous 24 h. One participant (9%) indicated that cough was not relevant to their experience. Another participant reported that only shortness of breath and cough were relevant. Some participants acknowledged that, even if a symptom did not apply to them, it could still be relevant to SAPH (or sarcoidosis) in general. Ten participants (91%) indicated that all 11 impact items were relevant to them, even if they had not experienced a given impact in the previous 7 days. The other participant (9%) indicated that only difficulty walking uphill and difficulty carrying things were relevant to them. All participants indicated that the oxygen use item was relevant.

All 10 participants who were asked for feedback on the PAH-SYMPACT™ instructions at the beginning of the questionnaire indicated that they were clear. Symptom and impact items were generally clear and understandable to all participants. Participants did not report that any key symptoms or impacts of SAPH were missing. One participant noted that “chest tightness” was similar to “chest pain.”

Two participants expressed general concerns. In grading symptoms, one participant felt it was unclear if they should consider the most severe episode in the previous 24 h or the average symptom severity, because their symptoms tended to vary over the course of 24 h. Another participant felt it was confusing to mention oxygen use in the instructions for the impact items.

Response options for the symptom and impact items were understood by most participants. Two participants reported difficulty deciding between specific response options (i.e., “mild” versus “moderate,” “moderate” versus “severe,” and “a little” versus “some”) because the distinction between the options was unclear.

**Fig. 1:**
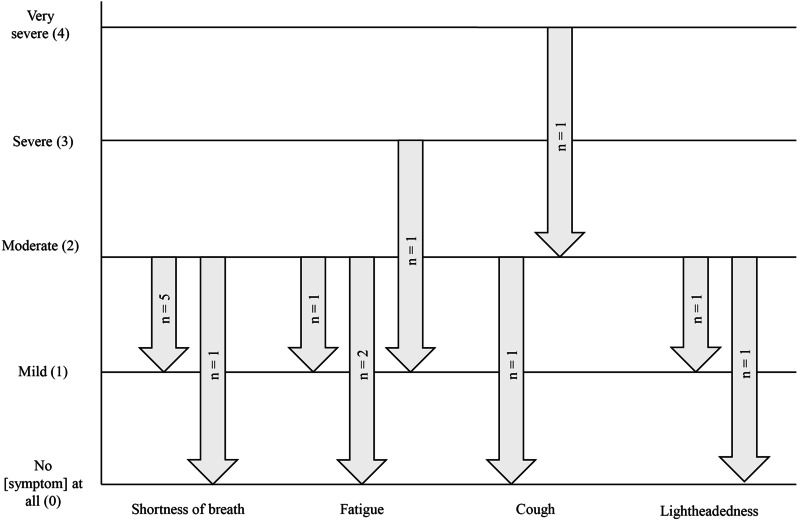
Meaningful improvement for symptom items. For each symptom item, only participants who scored the item as “(2)” or higher when completing the PAH-SYMPACT™ were included in the meaningful improvement discussions

For a subset of symptom and impact items, participants were asked to consider the response they gave when completing the PAH-SYMPACT™ and to indicate the minimum score change they would consider to represent a meaningful improvement. Participants were only asked to discuss meaningful improvement if their response to the item was “moderate” or worse. During discussions around meaningful improvement for the shortness of breath item, five (83%) of six participants indicated that a one-point score change would be meaningful (Fig. 1). For the fatigue item, three of four participants (75%) indicated that a two-point score change would be meaningful. Improvements considered meaningful in discussions surrounding the impact items are shown in Additional file 2: Figure S1. Overall, for the symptom and impact items that were discussed, the minimum score change considered to be meaningful improvement was one point in nine of 14 cases (64%) where participants' responses to the PAH-SYMPACT™ item indicated moderate severity (item score = 2). For participants whose PAH-SYMPACT™ responses indicated greater severity (item score = 3 or 4), a two- or three-point improvement was cited as representing meaningful improvement in all five cases (100%).

## Discussion

To our knowledge, this is the first qualitative interview study to assess patient experiences of SAPH symptoms and their impact. Most symptom and impact concepts emerged in the first two interview transcripts and saturation was achieved by the third set of interview transcripts (i.e., within the first eight interviews). Mirroring the qualitative work done during development of the PAH-SYMPACT [21], shortness of breath was endorsed by all 11 participants and was the most bothersome or severe symptom for a majority of participants. Walking, particularly uphill or up stairs, and performing daily activities were also problematic for all participants. Being dependent on others, negative impacts on family or parenting, difficulty walking uphill or up stairs, and negative impacts on relationships were among the most difficult impacts to cope with. Most participants indicated that the symptom and impact items included in the PAH-SYMPACT™ were relevant to their experiences of SAPH and sarcoidosis, even if they had not experienced them in the previous 24 h (symptoms) or 7 days (impacts). Participants also felt that the PAH-SYMPACT™ instructions and response options were clear and understandable, and that none of the key symptoms or impacts of sarcoidosis or SAPH were missing.

Other instruments used for assessing sarcoidosis include specific modules for assessing pulmonary symptoms. The SAT includes a module for assessing lung concerns [16, 29], and the KSQ also includes a lung module [30]. These lung-specific KSQ and SAT modules can be used to assess aspects of pulmonary sarcoidosis, and minimal clinically important differences have been established for both of them [16, 19]. However, the KSQ lung module only includes six items and does not specifically focus on SAPH [30], and the SAT lung module includes multiple items on symptoms such as cough that may be due to sarcoidosis rather than SAPH [29]. Neither the KSQ nor the SAT was developed for patients with PH. Moreover, in a trial of bosentan for SAPH, the SF-36 and St. George’s Respiratory Questionnaire failed to capture any improvement in quality of life, in spite of measurable improvements in hemodynamic parameters [31]. The PAH-SYMPACT™ is a relatively brief questionnaire that fulfils the need for an instrument to assess the specific symptoms and impacts of SAPH. It captures symptoms such as fatigue, lack of energy, and swelling in the ankles or legs and various physical and emotional impacts that are not included in the KSQ or SAT. Nine participants (82%) in the present study reported wheezing, which is not included in the PAH-SYMPACT™; however, wheezing is caused by fibrosis rather than by PH.

In general, participants indicated that a one-point improvement was meaningful if starting from a score representing moderate symptoms or impacts, whereas a two- or three-point improvement was considered as meaningful if starting from a score representing more severe symptoms or impacts. This suggests that the more severe a patient’s symptoms or impacts are, the greater improvement needs to be for patients to consider it meaningful. However, larger studies will be needed to establish usable meaningful change or responder thresholds.

The small sample size and recruitment of participants from only two sites in a single country limit generalizability of the present study findings to the overall population of patients with SAPH encountered in real-world clinical practice. The inclusion of two patients whose SAPH had been confirmed by echocardiography but not right heart catheterization is a further limitation. Finally, all of the participants were female. Compared to male patients, females with sarcoidosis are reported to have more difficulty in performing their daily activities, to be less physically active, and to have poorer physical health [32] and to be more greatly impacted emotionally [33]. The non-inclusion of male patients may therefore have skewed the severity or predominance of certain symptoms or impacts.

## Conclusions

Overall, the qualitative data collected in this study demonstrated that the PAH-SYMPACT™ was clear and well understood by study participants and that its items align with patient-reported symptoms and impacts of PH in patients with sarcoidosis. While our findings support utility of the PAH-SYMPACT™ in patients with SAPH, larger longitudinal studies are needed to confirm that the instrument is fit for use in this patient population and that it can be used to reliably detect temporal changes in patients’ symptom status and health-related quality of life.

## Supplementary Information

**Additional file 1:** Additional tables.

**Additional file 2:** Additional figure.

## Data Availability

The datasets used and/or analyzed during the current study are available from the corresponding author on reasonable request.

## Abbreviations

KSQ: King’s Sarcoidosis Questionnaire
PAH: Pulmonary arterial hypertension
PAH-SYMPACT™: Pulmonary Arterial Hypertension-Symptoms and Impact
PH: Pulmonary hypertension
PRO: Patient-reported outcome
SAPH: Sarcoidosis-associated pulmonary hypertension
SAT: Sarcoidosis Assessment Tool
SF-36: 36-Item Medical Outcomes Study Short Form Survey
