# Supplementary material for: Symptoms, impacts, and suitability of the Pulmonary Arterial Hypertension-Symptoms and Impact (PAH-SYMPACT™) questionnaire in patients with sarcoidosis-associated pulmonary hypertension (SAPH): a qualitative interview study
Source: BMC Pulm Med. 2021 Nov 12;21:365. doi: 10.1186/s12890-021-01694-1 (PMC8590341; doi:10.1186/s12890-021-01694-1)
Supplement: Supplementary file 1 — Additional file 1: Additional tables. [file 12890_2021_1694_MOESM1_ESM.pdf]

Supplemental Tables

Table S1 Additional details of the qualitative interviews

Qualitative interviews
Study organizational structure<br>• The study was conducted by the sponsor (Actelion) in collaboration with a contract research organization (Evidera)
Researchers<br>• Two female health outcomes research professionals (LS and SS), each with a master's degree and with training and experience in conducting qualitative interviews
Process<br>• The study sites provided the researchers with contact information for eligible patients who were willing to participate<br>• One of the researchers (SS) then contacted potential participants by telephone to schedule the interviews<br>• At the beginning of the interview, the researcher conducting the interview (LS or SS) introduced herself by name to participants, and provided her employer's name (Evidera) and the reason for doing the interviews<br>• Audio files were transcribed verbatim by a transcription vendor, Gordon Transcripts<br>• The final "cleaned" transcripts were not returned to participants for comment or correction
Analysis<br>• A coding framework was developed by one researcher (LS) using the main concepts included in the interview guide<br>• The coding framework was revised based on findings from the early interviews<br>• Using the resulting coding dictionary, two researchers (LS and SS) independently coded one transcript<br>• The dual-coded transcript was reviewed to confirm that the researchers had captured all relevant responses and were interpreting and using codes consistently and as intended. The remaining transcripts were then coded by one of the researchers (SS) and the other researcher (LS) checked the quality of the coded transcripts<br>• All quantitative data were collected using DataFax (DF/Net Research, Inc., Seattle, WA), an optical character recognition software package that is compliant with Part 11 of Title 21 of the US Food and Drug Administration Code of Federal Regulations

Table S2 Saturation grid for symptom concepts

	Set of transcripts				
	First (transcripts 1 and 2) n	Second (transcripts 3 to 5) n	Third (transcripts 6 to 8) n	Fourth (transcripts 9 to 11) n	All transcripts (N=11) n (%)
Shortness of breath	2	3	3	3	11 (100)
Swelling in ankles or legs	2	3	3	3	11 (100)
Fatigue	2	2	3	3	10 (91)
Lack of energy	1	2	3	3	9 (82)
Cough	1	2	3	3	9 (82)
Rapid heartbeat	2	2	3	2	9 (82)
Wheezing	2	1	3	3	9 (82)
Skin issues	2	2	3	2	9 (82)
Chest pain	1	1	3	3	8 (73)
Lightheadedness	2	1	2	3	8 (73)
Eye issues	2	1	3	2	8 (73)
Non-chest pain	1	1	2	3	7 (64)
Chest tightness	1	2	1	2	6 (55)
Swelling in stomach area	1	1	1	0	3 (27)
Balance issues	1	0	0	0	1 (9)
Legs feeling like they will "give out"	1	0	0	0	1 (9)
Numbness in fingers	1	0	0	0	1 (9)
Problems urinating (attributed to sarcoidosis in kidneys)	1	0	0	0	1 (9)
Seizures and Bell's palsy symptoms (attributed to sarcoidosis in the brain and stress)	1	0	0	0	1 (9)
Swelling in hands	1	0	0	0	1 (9)
Heart palpitations	0	2	3	2	7 (64)
Headache	0	1	2	0	3 (27)
Heavy feeling in the chest or pressure on the chest (different than tightness or pain)	0	1	0	0	1 (9)
Weakness (general)[a]	0	0	1	0	1 (9)
Weakness and pain in hands[a]	0	0	0	1	1 (9)
Left side of body feels different (feels "not good")[a]	0	0	0	1	1 (9)
Congestion; lump in throat (needing to clear throat)[a]	0	0	0	1	1 (9)

Numbers represent the number of participants within each transcript group who raised or endorsed a particular symptom concept spontaneously or upon probing.

[a]It was judged that these concepts overlapped with concepts that had been raised or endorsed by earlier transcript groups or were likely related to other health conditions.

Table S3 Saturation grid for impact concepts

	Set of transcripts				
	First (transcripts 1 and 2)	Second (transcripts 3 to 5)	Third (transcripts 6 to 8)	Fourth (transcripts 9 to 11)	All transcripts (N=11)
	n	n	n	n	n (%)
Difficulty walking up stairs	2	3	3	3	11 (100)
Ability to walk (general)	2	2	3	3	10 (91)
Carrying things	2	2	3	3	10 (91)
Housework	2	2	3	3	10 (91)
Difficulty with hills	1	2	3	3	9 (82)
Hobbies	1	2	3	3	9 (82)
Oxygen use	2	2	2	2	8 (73)
Work/school/volunteering	2	1	2	3	8 (73)
Other daily activities	2	2	1	2	7 (64)
Self-care activities	1	1	2	3	7 (64)
Mental functioning	2	1	2	2	7 (64)
Dependence on others	1	2	2	2	7 (64)
Relationships	1	2	2	2	7 (64)
Sleep impacted	1	3	1	2	7 (64)
Running or exercise	1	2	2	1	6 (55)
Walking quickly	2	2	1	0	5 (45)
Walking slowly	2	1	1	1	5 (45)
Feeling frustrated or angry	2	1	1	1	5 (45)
Feeling worried or anxiety	1	1	1	1	4 (36)
Feeling sad or depression	1	1	1	1	4 (36)
Driving	1	1	0	1	3 (27)
Feeling embarrassed	1	0	1	1	3 (27)
Parenting or family impact	2	0	1	0	3 (27)
Running errands/shopping	1	0	0	1	2 (18)
Clothing or shoe fit from swelling	1	0	0	1	2 (18)
Impact of knowing you will never be healthy	0	1	0	0	1 (9)
Talking or singing impacted (changed voice, difficulty talking with exertion)	0	0	1	1	2 (18)
Bone issues (fragile, "dead bone")	0	0	1	1	2 (18)
Social activities	0	0	1	0	1 (9)
Weight loss	0	0	1	0	1 (9)
Incontinence	0	0	1	0	1 (9)

Numbers represent the number of participants within each transcript group who raised or endorsed a particular impact concept spontaneously or upon probing.
