# Supplementary figures and images for: Symptoms, impacts, and suitability of the Pulmonary Arterial Hypertension-Symptoms and Impact (PAH-SYMPACT™) questionnaire in patients with sarcoidosis-associated pulmonary hypertension (SAPH): a qualitative interview study
Source: BMC Pulm Med. 2021 Nov 12;21:365. doi: 10.1186/s12890-021-01694-1 (PMC8590341; doi:10.1186/s12890-021-01694-1)

Figure S1

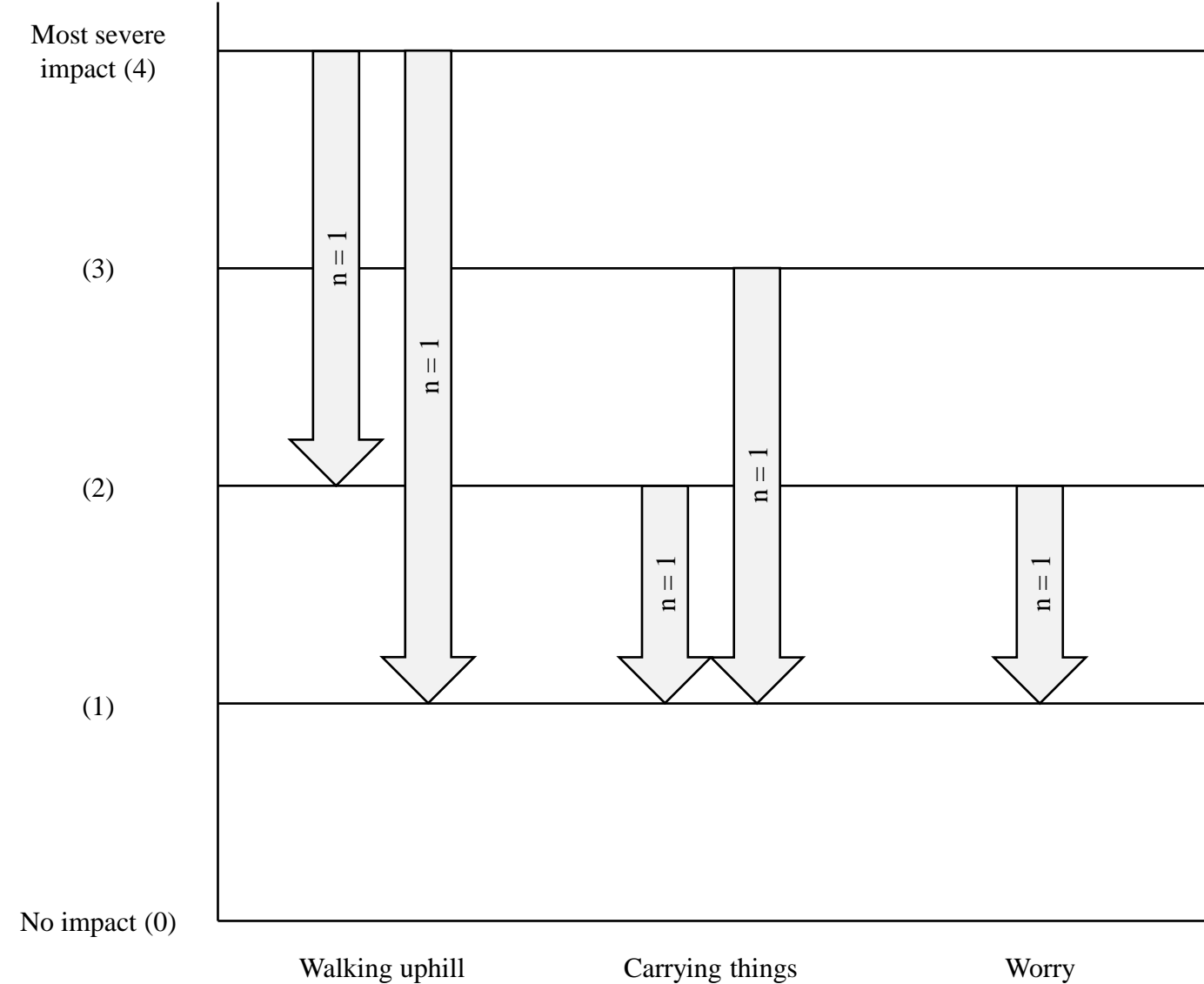

Supplement: Supplementary file 2 — Additional file 2: Additional figure. [file 12890_2021_1694_MOESM2_ESM.pdf]
